# Optimization of the fermentation media and growth conditions of *Bacillus velezensis* BHZ-29 using a Plackett–Burman design experiment combined with response surface methodology

**DOI:** 10.3389/fmicb.2024.1355369

**Published:** 2024-04-22

**Authors:** YingWu Shi, XinXiang Niu, HongMei Yang, Ming Chu, Ning Wang, HuiFang Bao, FaQiang Zhan, Rong Yang, Kai Lou

**Affiliations:** ^1^Institute of Microbiology, Xinjiang Academy of Agricultural Sciences, Ürümqi, China; ^2^Xinjiang Laboratory of Special Environmental Microbiology, Ürümqi, China; ^3^Key Laboratory of Agricultural Environment in Northwest Oasis of Ministry of Agriculture and Countryside, Ürümqi, China; ^4^Institute of Soil Fertilizer and Agricultural Water Conservation, Xinjiang Academy of Agricultural Sciences, Ürümqi, China

**Keywords:** optimization, fermentation media, fermentation conditions, *Bacillus velezensis* BHZ-29, Plackett–Burman design, response surface methodology

## Abstract

**Introduction:**

*Bacillus velezensis* occurs extensively in the soil environment. It produces a range of antimicrobial compounds that play an important role in the field of biological control. However, during the actual application process it is often affected by factors such as the medium formulation and fermentation conditions, and therefore biocontrol measures often do not achieve their expected outcomes.

**Methods:**

In this study, the *B. velezensis* BHZ-29 strain was used as the research object. The carbon and nitrogen sources, and inorganic salts that affect the number of viable bacteria and antibacterial potency of *B. velezensis* BHZ-29, were screened by a single factor test. A Plackett–Burman design experiment was conducted to determine the significant factors affecting the number of viable bacteria and antibacterial potency, and a Box–Behnken design experiment was used to obtain the optimal growth of *B. velezensis* BHZ-29. The medium formula that produced the highest number of viable bacteria and most antibacterial substances was determined. The initial pH, temperature, amount of inoculant, liquid volume, shaking speed, and culture time were determined by a single factor test. The factors that had a significant influence on the number of viable bacteria of *B. velezensis* BHZ-29 were selected by an orthogonal test. A Box–Behnken design experiment was conducted to obtain the optimal fermentation conditions, and highest number of viable bacteria and antibacterial titer.

**Results:**

Molasses, peptone, and magnesium sulfate had significant effects on the viable count and antibacterial titer of *B. velezensis* BHZ-29. The viable count of *B. velezensis* BHZ-29 increased from 7.83 × 10^9^ to 2.17 × 10^10^ CFU/mL, and the antibacterial titer increased from 111.67 to 153.13 mm/mL when the optimal media were used. The optimal fermentation conditions for *B. velezensis* BHZ-29 were as follows: temperature 25.57°C, pH 7.23, culture time 95.90 h, rotation speed 160 rpm, amount of inoculant 2%, and liquid volume 100 ml. After the optimization of fermentation conditions, the number of viable bacteria increased to 3.39 × 10^10^ CFU/mL, and the bacteriostatic titer increased to 158.85 mm/ml.

The plant height and leaf number of cotton plants treated with BHZ-29 fermentation broth were higher than those of cotton inoculated with *Verticillium dahliae*. The number of bacteria was 1.15 × 10^7^ CFU/g, and the number of fungi was 1.60 × 10^5^ spores/g. The disease index of the cotton seedlings treated with the optimized fermentation broth was 2.2, and a control effect of 93.8% was achieved. *B. velezensis* BHZ-29 could reduce the disease index of cotton *Verticillium* wilt and had a controlling effect on the disease. The best effect was achieved in the treatment group with an inoculation concentration of 2 × 10^8^ CFU/ml, the disease index was 14.50, and a control effect of 84.18% was achieved.

**Discussion:**

The fermentation process parameters of the number of viable bacteria and antibacterial titer by strain *B. velezensis* BHZ-29 were optimized to lay a foundation for the practical production and application of strain *B. velezensis* BHZ-29 in agriculture.

## 1 Introduction

Xinjiang is the largest cotton production base in China; however, cotton *Verticillium* wilt is an important disease that has seriously hindered the development of the Xinjiang cotton industry (Xue et al., 2013). The occurrence of cotton *Verticillium* wilt in Xinjiang is becoming increasingly serious due to unfavorable factors, such as continuous cropping, returning cotton stalk to the field, and the poor disease resistance of varieties, resulting in huge economic losses (Ma, 2007; Liu et al., 2015a,b). Biological control has become a hot spot in cotton *Verticillium* wilt research because of its advantages of producing no pollution or residues, not harming humans and livestock, and not causing pathogenic fungus resistance (Ma et al., 1997). The use of bacteria for the biocontrol of cotton *Verticillium* wilt is a major focus in biological control research. Antagonistic bacteria are known for their variety, wide distribution, rapid growth, strong stress resistance, and production of many secondary metabolites, and are therefore the main flora used for the biological control of cotton *Verticillium* wilt (Liu J. et al., 2023; Liu L. et al., 2023). It is therefore of great significance to develop environmentally friendly and efficient biocontrol agents for the biological control of important soil-borne diseases of cotton and to promote the green production of cotton.

*Bacillus velezensis* has great potential in the prevention and control of plant diseases, but some strains have only a single function, and at the same time, there is also a lack of development and utilization of diversified functions of strains. Additionally, the antibacterial activity of strains is usually related to their biomass, the yield of active substances, and colonization ability (Zhang H. et al., 2023; Zhang L. et al., 2023). The optimization of fermentation conditions would create a suitable environment for the growth of various strains, thus promoting growth and increasing the content of active antibacterial substances in the fermentation broth of strains, both of which will improve the biocontrol potential of these strains (Li R. Q. et al., 2018; Li X. Y. et al., 2018).

The growth and reproduction of microorganisms are inseparable from the medium, and the composition of the medium is essential for the growth, development, metabolism, and product accumulation of microorganisms (Ahsan et al., 2017). Due to the use of unsuitable culture media, the number of viable bacteria in the fermentation process is often low, and few antibacterial substances are secreted, which diminishes the biocontrol potential for these microorganisms against diseases. The selection and proportional composition of microbial media components are therefore particularly important. Based on a single-factor test and the central composite design of response surface methodology, the optimum fermentation medium for *Bacillus amyloliquefaciens* antagonistic substance was determined, containing 15 gm/L of semolina flour, 12.5 gm/L of beef extract, and 0.5 gm/L of magnesium sulfate, which inhibited the fungal growth by 91% (Ahsan et al., 2022), and a new optimal formulation was determined as follows: 10% silica, 40% soybean oil, 8% ST, 1.51% AEC-9NA, 2.36% glycol, 0.08% sodium alginate, and 2% SY-6535 (Zhang H. et al., 2023; Zhang L. et al., 2023). Under optimized conditions, the viable count of *B. amyloliquefaciens* Lx − 11 SE was up to 9.3 × 10^8^ colony forming units (cfu) per ml. The results showed that the regression equation model had satisfactory accuracy in predicting the viable count of *B. amyloliquefaciens* Lx-11 SE. Under these optimal culture conditions, the titer of antifungal substances produced by the *Paenibacillus polymyxa* DS-R5 was 77.6% higher than that under the initial culture conditions. Response surface methodology can be well applied the optimization of culture conditions for antifungal substance, which lays the foundation for further research on the DS-R5 strain (Sa et al., 2022). Different strains require different nutrients, and the optimal medium for *B. velezensis* BHZ-29 has not been reported.

Different strains have different physiological characteristics and habitat preferences. The optimal fermentation temperature, fermentation time, rotation speed, carbon source, nitrogen source, and inorganic salts are different. Under the optimal parameters level (frequency of 40 kHz, power density of 40 W/L, time of 17.5 min) of *Bacillus amyloliquefaciens*, peptide content of SSF soybean meal in ultrasonic treatment group reached 150.68 mg/g, which increased by 13.10% compared to the control (Wang et al., 2021). The optimal carbon source, nitrogen source, and precursor for *Bacillus siamensis* CAU83 producing gamma-PGA were 30 g/L lactose, 5 g/L yeast extract, and 60 g/L L-sodium glutamate, respectively. The optimal fermentation conditions were 37°C and pH 7.0. The yield of gamma-polyglycolic acid (PGA) increased by 260% from 8.4 g/L before optimization to 30.1 g/L after optimization (Lin et al., 2022). The average amount of dried extract produced by *Bacillus velezensis* RP137 in the optimum conditions was 131.1 mg/L and the best response was 71.45%, which is more than 28-fold better than the pre-optimized conditions (Pournejati et al., 2020). Therefore, determining the appropriate fermentation conditions for different strains is a key step in the production of functional strains and will enable their biocontrol functions to be realized.

Different microorganisms have different nutritional needs. Before the industrial production of microorganisms, it is necessary to optimize the fermentation medium and culture conditions. The methods used to optimize the fermentation medium include a single factor experiment (Tian et al., 2014), orthogonal test (Tu et al., 2015), uniform design (Ruan et al., 2012), Plackett–Burman design (Quan et al., 2015), Box–Behnken design (Shang et al., 2013; Quan et al., 2015), and central composite design (Lima et al., 2011). The optimization of different microbial fermentation media has been achieved previously using Plackett–Burman design, Box–Behnken design, or central composite design experiments. Using response surface methodology (RSM), the optimal medium or culture conditions can be quickly and effectively determined.

*Bacillus velezensis* BHZ-29 was isolated from cotton plants in our laboratory (Zhang et al., 2018). The results of an indoor bioassay showed that the strain had a strong control effect on cotton *Verticillium* wilt, jujube black spot, melon postharvest rot, and grape rot, and had some value in the development of microbial pesticide products. In this study, *B. velezensis* BHZ-29 was used as the research object, and a Plackett–Burman design experiment was conducted to optimize the components of a shaken flask formula for the nutrient broth fermentation medium to screen out the significant influencing factors. Then, the steepest ascent test and Box–Behnken design experiment were conducted, enabling a nonlinear equation to be fitted between the significant factors and spore yield, and the fermentation sporulation medium formula of the strain was optimized. Taking the number of viable bacteria as the index, the range of fermentation conditions was determined by a single factor test. An orthogonal test was used to select the significant factors affecting the viable count of *B. velezensis* BHZ-29. A Box–Behnken design experiment was then conducted according to the orthogonal test results, and the simulation fitting equation was established based on the test results. The optimal fermentation conditions were obtained, which resulted in improved viable bacteria numbers. Validation of fermentation processes was carried out in a 1,000 L fermentor to provide support for the expansion of production and industrialization of the BHZ-29 strain.

## 2 Materials and methods

### 2.1 Microorganisms and media

*Bacillus velezensis* BHZ-29 was isolated, purified, and preserved by the laboratory of Institute of Microbial Application, Xinjiang Academy of Agricultural Sciences (Zhang et al., 2018). *Verticillium dahlia* 2015–007 was isolated from diseased plants in the Shihezi cotton area and preserved with moderate to strong pathogenicity. Potato dextrose water medium (PDB) was prepared as follows: potato extract 6.0 g, glucose 20.0 g, distilled water 1,000 mL, pH 5.6 ± 0.2. Nutrient broth medium (NB) was prepared as follows: peptone 10.0 g, beef extract 3.0 g, sodium chloride 5.0 g, distilled water 1,000 mL, pH 7.2 ± 0.2.

### 2.2 Preparation of inoculum and flask cultures

The preserved *B. velezensis* BHZ-29 was streaked and inoculated on nutrient agar (NA) medium and cultured at 32°C for 48 h. The volume of liquid medium was 100 mL per 500 mL Erlenmeyer flask. A single colony of *B. velezensis* BHZ-29 was selected and inoculated into 100 mL NB liquid medium, and the seed liquid was obtained by shaking a culture at 32°C and 180 rpm for 12 h (Hassan et al., 2011). *Fusarium oxysporum* cakes were placed on a potato dextrose agar (PDA) medium, and cultured at 28°C for 5 days. The liquid volume of PDB liquid medium was 200 mL per 500 mL Erlenmeyer flask. Small fungal cakes (6 mm) were prepared by a punching method. Each bottle was inoculated with 15 fungal cakes, and cultured at 28°C and 160 rpm for 5 days. The filtrate was filtered through a sterile gauze, and the filtrate was collected and used as the spore suspension of *F. oxysporum* (Shim et al., 2013).Erlenmeyer flask.

### 2.3 Fermentation medium screening

#### 2.3.1 Screening of significant variables by a Plackett–Burman design experiment

Fermentation medium composition was based on NB medium. Glucose, sucrose, lactose, fructose, maltose, soluble starch, starch, molasses, and Indian meal, each with a concentration of 10 g/L, were selected to replace the carbon source in the basic fermentation medium, while the other components remained unchanged. Beef extract, peptone, yeast extract, urea, ammonium nitrate, ammonium sulfate, sodium nitrate, soybean powder, and cottonseed meal, each with a concentration of 10 g/L, were selected to replace peptone and beef powder in the basic fermentation medium, while the other components remained unchanged. Potassium chloride, calcium chloride, magnesium sulfate, zinc sulfate, ferrous sulfate, calcium carbonate, manganese sulfate, and dipotassium hydrogen phosphate, each with a concentration of 5 g/L, were selected to replace sodium chloride in the basic fermentation medium, while the other components remained unchanged. Experiments were carried in triplicates. A 0.5 ml portion of fermentation broth was added to 4.5 ml of 0.03% Tween 80, the solution was successively diluted 10 times, and 100 μl was evenly coated on NA medium. It was cultured at 32°C for 2 days, and the number of viable bacteria was counted. Then, 100 μl of *F. oxysporum* spore suspension was evenly coated on PDA medium where it was left to stand for 30 min. Four 6 mm diameter holes were punched equidistantly on the medium using a sterile puncher, and 100 μl of fermentation broth was injected, respectively. The fermentation broth was cultured at 28°C for 4 days, the diameter of the inhibition zone was measured, and the bacteriostatic titer was calculated. The bacteriostatic titer was defined as the diameter of the 1 mm inhibition zone produced by each 1 ml fermentation broth. Bacteriostatic titer (mm/mL) = (X − Y)/V × 1,000, where X is the diameter of the inhibition zone (mm), Y is the diameter of the puncher (6 mm), and V is the volume of bacterial suspension (μL).

The Plackett–Burman design experimental was conducted using DesignExpert 10.0 software to further screen the condition factors determined by the single factor experiment. Each factor was divided into low and high levels, and a total of 12 experiments were conducted.

#### 2.3.2 The steepest climbing test and Box–Behnken design experiment

According to the proportion of important factor effects determined by the results of the Plackett–Burman design experiment, the gradient direction and range of test value changes were set, and the optimal optimization conditions were selected as the central point of the RSM results. A three-level RSM analysis was conducted on three significant influencing factors using a Box–Behnken design experiment. The data were fitted by a quadratic regression to obtain a quadratic equation with interaction and square terms. The variance analysis of the regression equation was carried out to evaluate the influence of each factor and its interaction on the response value. At the same time, the level of each factor when the response value was largest was obtained, and the optimal conditions determined by the regression model were verified. The experimental scheme and regression analysis were designed using the Design Expert 10.0 software.

### 2.4 Optimization of the fermentation conditions

#### 2.4.1 Optimization of the fermentation conditions of *Bacillus velezensis* BHZ-29 by a single factor test

The culture conditions were optimized using the optimized medium. The volume of liquid in a 500 ml conical flask was 100 ml. The single factor test had the basic culture conditions of: initial pH 7.2, amount of inoculant 1%, temperature 32°C, and rotation speed 180 rpm for 72 h. The number of viable bacteria and antibacterial titer of the *B. velezensis* BHZ-29 fermentation broth were determined, and the antibacterial titer was measured by the diameter of the inhibition zone.

#### 2.4.2 Optimization of the fermentation conditions of *Bacillus velezensis* BHZ-29 by an orthogonal test and RSM

The orthogonal test was conducted under the range of conditions obtained by the single factor test. Assuming that there was an interaction between the factors, an orthogonal test of six factors and three levels L18 (36) was designed (Table 1). Based on the results of the orthogonal test, the temperature (A), pH (C), and culture time (F) that had a significant effect on the number of viable *B. velezensis* BHZ-29 bacteria were selected for optimization, and a Box–Behnken design experiment was conducted with the number of viable bacteria as the response value.

**Table 1:** Fermentation factor and level of BHZ-29.

Variables	Code	Level		
		−1	0	1
Temperature	A	24	26	28
Rotational speed	B	160	180	200
Initial pH	C	7.0	7.2	7.4
Inoculation amount	D	1	2	3
Broth content	E	50	100	150
Time	F	48	72	96

### 2.5 Shaken flask fermentation verification test

The optimized medium was adjusted as follows: molasses 20.38 g/L, peptone 19.40 g/L, and magnesium sulfate 3.56 g/L. The number of viable bacteria in the fermentation broth of *B. velezensis* BHZ-29 was determined, and the fitting degree between the actual and predicted values of the number of viable bacteria was verified. The optimized medium was adjusted to 19.44 g/L molasses, 20.42 g/L peptone, and 3.51 g/L magnesium sulfate. The antibacterial titer of the fermentation broth of *B. velezensis* BHZ-29 was determined to verify the fitting degree between the actual and predicted values of the antibacterial titer. The experiment was repeated five times.

### 2.6 The control effect of the *Bacillus velezensis* BHZ-29 optimized fermentation culture on cotton *Verticillium* wilt

The soil was sterilized at 180°C for 3 h, and Xinluzao 36 cotton seeds were planted with six plants per pot. When the cotton seedlings grew to two leaves and one heart, the fermentation broth of *V. dahliae* with a concentration of 2 × 10^7^ spores/mL was inoculated by the root injury perfusion method at 20 mL/plant. After 7 days, the fermentation broths with different concentrations of antagonistic bacteria were inoculated by a root irrigation method at 20 mL/plant. Three treatments were established: (1) *V. dahliae* fermentation broth + strain *B. velezensis* BHZ-29 fermentation broth; (2) *V. dahliae* fermentation broth; and (3) clear water control. The inoculation concentration of the antagonistic bacteria fermentation broth was set to three levels of 2 × 10^8^, 2 × 10^6^, or 2 × 10^5^ CFU/mL. A pot disease prevention test for each of the three pot treatments was conducted with three replicates. After 60 days of cotton seedling growth, the plants were graded according to the classification method of Zhu et al. (2013): grade 0: healthy plant; grade 1: 1–2 cotyledons were diseased; grade 2: 1 true leaf diseased; grade 3: more than 2 true leaves diseased or fallen off; and grade 4: all leaves fallen off or the apex withered. The disease index and control effect of cotton *Verticillium* wilt were calculated according to the grading of the cotton *Verticillium* wilt disease index. Disease index = ∑(grade value × plant number)/(highest grade value × total plant number) × 100; control effect (%) = (control disease index-treatment disease index)/control disease index × 100.

### 2.7 Statistical analysis

The Plackett–Burman design experiment, Box–Behnken design experiment, and RSM were conducted using DesignExpert12 software. The results of the experiments were statistically analyzed using SPSS 22.0 software, and the least significant difference method was used to test for significant differences.

## 3 Results

### 3.1 Selection of carbon, nitrogen, and mineral sources

**Figure 1:**
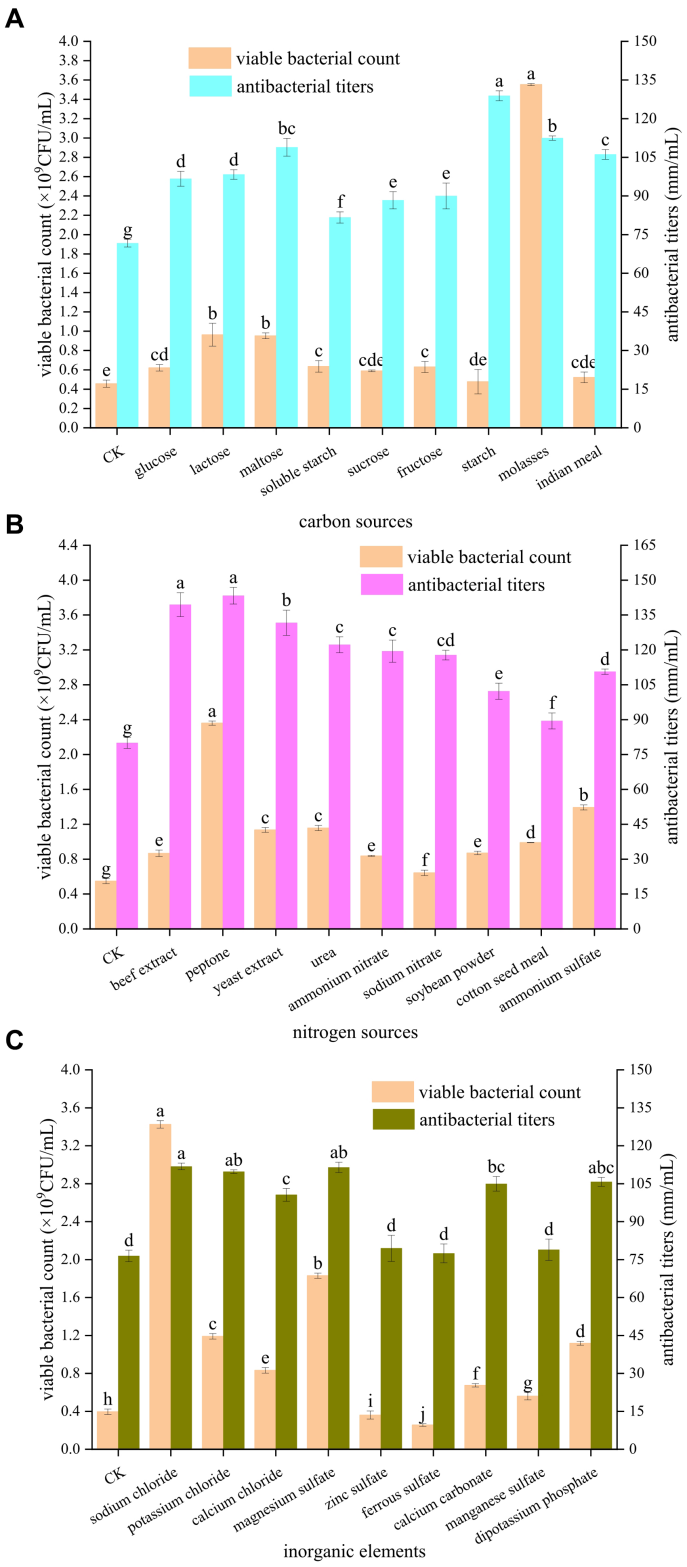
Effects of different carbon sources **(A)**, nitrogen sources **(B)**, and inorganic elements **(C)** on the fermentation of strain BHZ-29. Different lowercase letters mean significant difference. (*P* < 0.05).

As shown in Figure 1A, the different carbon sources had different promotional effects on the number of viable bacteria and antibacterial potency of *B. velezensis* BHZ-29. The number of viable bacteria in the fermentation broth was highest (3.55 × 10^9^ CFU/mL) when molasses were used as the carbon source. When glucose, lactose, maltose, soluble starch, fructose, and molasses were used as carbon sources, the numbers of viable *B. velezensis* BHZ-29 bacteria were significantly different from that of the control. When sucrose, starch, and corn flour were used as carbon sources, the numbers of viable *B. velezensis* BHZ-29 bacteria were not significantly different from that of the control. The antibacterial titer was highest (128.89 mm/mL) when starch was used as the carbon source, but the number of viable bacteria was lower and there was no significant difference with the control. For molasses, maltose, and corn flour the antibacterial titers were 112.44, 108.89, and 106.11 mm/mL, respectively. Therefore, molasses and maltose were selected as the carbon source of the medium.

As shown in Figure 1B, the different nitrogen sources also had different effects on the number of viable bacteria and antibacterial titer of *B. velezensis* BHZ-29. Among the organic nitrogen sources, peptone and yeast extract were the most suitable for the growth and reproduction of *B. velezensis* BHZ-29, producing numbers of viable bacteria of 2.36 × 10^9^ and 1.14 × 10^9^ CFU/mL, respectively. Among the inorganic nitrogen sources, ammonium sulfate and urea were the most suitable for the growth and reproduction of *B. velezensis* BHZ-29, producing numbers of viable bacteria of 1.39 × 10^9^ and 1.16 × 10^9^ CFU/mL, respectively. For organic nitrogen sources, the fermentation broth had the best inhibitory effect on the target pathogen when peptone and beef extract were used, and the difference between the two nitrogen sources was not significant. The bacteriostatic titers were 143.33 and 139.44 mm/mL, respectively. For inorganic nitrogen sources, the fermentation broth had the best inhibitory effect on the target pathogen when urea and ammonium nitrate were used, and the difference between the two nitrogen sources was not significant. The antibacterial titers were 122.22 and 119.44 mm/ml, respectively. Based on the effects of different nitrogen sources on the number of viable bacteria and bacteriostatic titer of *B. velezensis* BHZ-29, peptone and ammonium sulfate were selected as the nitrogen sources of the medium.

As shown in Figure 1C, the different inorganic salts also had different effects on the number of viable bacteria and the antibacterial activity of *B. velezensis* BHZ-29. Ferrous sulfate and zinc sulfate had a significant inhibitory effect on the number of viable *B. velezensis* BHZ-29 bacteria. Sodium chloride, calcium chloride, magnesium sulfate, calcium carbonate, manganese sulfate, dipotassium hydrogen phosphate, and potassium chloride had significant promotional effects on the number of viable *B. velezensis* BHZ-29 bacteria. The number of viable cells produced in the presence of sodium chloride, magnesium sulfate, and potassium chloride were 3.43 × 10^9^, 1.83 × 10^9^, and 1.19 × 10^9^ CFU/mL, respectively. All inorganic salts promoted antibacterial activity. The fermentation broth with sodium chloride, magnesium sulfate, and potassium chloride as inorganic salts had a stronger antibacterial effect on the target pathogen, with antibacterial titers of 111.82, 111.46, and 109.76 mm/ml, respectively. Therefore, sodium chloride and magnesium sulfate were selected as inorganic salts for incorporation in the medium.

### 3.2 Screening of significant variables by the Plackett–Burman design experiment

A Plackett–Burman design experiment (*N* = 12) was conducted. According to the results of the single factor test, molasses (X1), maltose (X2), peptone (X3), ammonium sulfate (X4), sodium chloride (X5), and magnesium sulfate (X6) were selected as six factors to investigate. The high and low levels of these variables were selected, and the number of viable bacteria (Y1) and the bacteriostatic titer (Y2) were used as experimental indexes. The experimental design and results are shown in Table 2. A multiple linear regression equation was obtained by a regression analysis of the data in Table 2: Y1 = 1.039 + 0.165 × 1 + 0.080 × 2 + 0.390 × 3 + 0.192 × 4-0.012 × 5 + 0.295 × 6; Y2 = 139.569 + 0.779 × 1-0.234 × 2 + 1.371 × 3 + 0.562 × 4 + 0.617 × 5 + 0.941 × 6.

**Table 2:** The design and results of Plackett–Burman experiment.

Run numbers	X1	X2	X3	X4	X5	X6	Y1	Y2
							(10^10^ CFU/mL)	**(mm/mL)**
1	1	1	1	−1	1	1	2.24	140.24
2	1	−1	−1	−1	1	1	0.63	135.78
3	−1	1	1	−1	1	−1	0.57	141.89
4	1	1	−1	1	−1	−1	0.84	138.13
5	1	1	−1	1	1	−1	0.74	138.60
6	−1	−1	−1	−1	−1	−1	0.36	139.80
7	−1	−1	1	1	1	−1	1.23	137.74
8	−1	1	−1	−1	-1	-1	0.56	137.05
9	-1	1	1	1	-1	1	1.77	141.06
10	1	-1	1	1	-1	1	2.06	143.43
11	-1	-1	-1	1	1	1	0.76	139.39
12	1	-1	1	-1	-1	-1	0.72	141.72

The effects of various factors on the number of *B. velezensis* BHZ-29 colonies were analyzed. The results are shown in Table 2. The effects of these factors on the antibacterial titer of *B. velezensis* BHZ-29 are also shown in Table 2. The importance of the six factors on the colony number of *B. velezensis* BHZ-29 was peptone > magnesium sulfate > ammonium sulfate > molasses > maltose > sodium chloride. Peptone and magnesium sulfate had a significant effect on the colony number at the 0.05 level. The importance of the six factors on the antibacterial titer of *B. velezensis* BHZ-29 was peptone > magnesium sulfate > molasses > ammonium sulfate > sodium chloride > maltose. After a comprehensive consideration, molasses was selected as the carbon source, peptone as the nitrogen source, and magnesium sulfate as the inorganic salt for further optimization.

### 3.3 Determination of the central point levels by the steepest ascent experiments and determination of the optimal medium

According to Table 3, with increased amounts of molasses, peptone, and magnesium sulfate, the number of viable bacteria and bacteriostatic titer displayed a trend of first increasing and then decreasing. When the molasses concentration was 20 g/L, peptone concentration was 20 g/L, and magnesium sulfate concentration was 3.5 g/L, the number of viable bacteria and bacteriostatic titer reached their maximum values. Therefore, the proportional composition of the treatment 3 fermentation medium was used as the central point of the RSM for further optimization.

**Table 3:** Regression analysis of experimental results based on Plackett–Burman design.

Factors		Levels		T	P	Order
Symbols	Variables	low(−1)	high(1)			
X_1_	Molasses (g/L)	5	10	2.643	0.046	3
X_2_	Maltose (g/L)	5	10	−0.792	0.464	6
X_3_	Peptone (g/L)	5	10	4.635	0.006	1
X_4_	Ammonium sulfate (g/L)	5	10	1.902	0.116	5
X_5_	Sodium chloride (g/L)	0.5	2.5	2.088	0.091	4
X_6_	Magnesium sulfate (g/L)	0.5	2.5	3.181	0.025	2

Based on the results of the steepest ascent test, three concentrations of molasses (15, 20, and 25 g/L), peptone (15, 20, and 25 g/L) and magnesium sulfate (3, 3.5, and 4 g/L) were established with the proportional composition of the treatment 3 fermentation medium as the central point. A regression analysis was performed on the data in Supplementary Table S1, and the following quadratic polynomial equation was obtained: Y1 = 1.900–0.240 × 1 + 0.150 × 3-0.063×6-0.160X1X3–0.0170X1X6 + 0.092X3X6–0.430 × 12-0.440 × 32-0.490×62 (*R*^2^ = 0.9952, *R*^2^ adjustment = 0.8976); Y2 = 151.09–3.57 × 1 + 2.41 × 3-0.77 × 6 + 0.10X1X3–1.53X1X6 + 2.48X3X6–5.31 × 12-4.45 × 32-5.31 × 62 (R^2^ = 0.9405, *R*^2^ adjustment = 0.8640).

The results of the variance analysis of the regression model with the number of colonies as the response value are shown in Table 4, and the results of the variance analysis of the regression model with the antibacterial potency as the response value are shown in Supplementary Table S2. The results showed that at the 0.05 level, the regression of the two models was significant, and the first (X1 and X3) and second (X1^2^, X3^2^, and X6^2^) terms had significant effects on the experiment.

**Table 4:** The design scheme and test results of the Box–Behnken design.

Run numbers	*X*_1_	*X*_3_	*X*_6_	*Y*_1_	*Y*_2_
				(10^10^ CFU/mL)	(mm/mL)
1	−1	−1	0	0.91	142.08
2	−1	1	0	1.54	146.10
3	1	1	0	0.84	140.77
4	0	0	0	1.99	153.97
5	1	−1	0	0.84	136.35
6	0	−1	1	0.76	135.31
7	0	−1	−1	0.93	142.28
8	1	0	1	0.54	134.21
9	0	0	0	1.85	151.18
10	0	1	1	1.21	145.68
11	−1	0	1	1.16	146.00
12	0	0	0	1.66	147.51
13	−1	0	1	1.16	144.03
14	1	0	−1	0.84	138.35
15	0	0	0	1.89	149.60
16	0	0	0	2.11	153.17
17	0	1	−1	1.01	142.74

**Figure 2:**
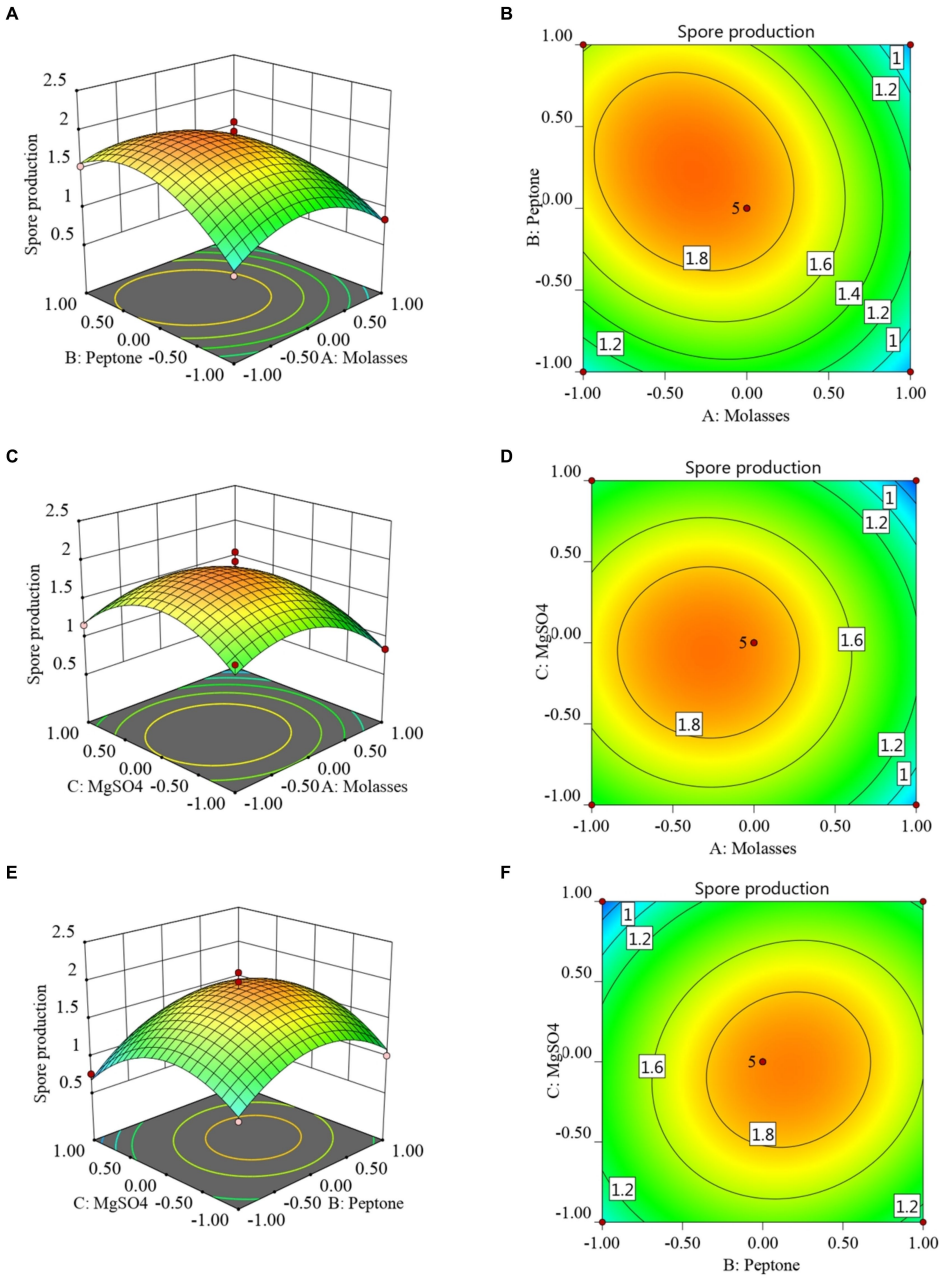
Response surface **(A, C, E)** and contour lines **(B, D, F)** of the interaction among three factors of medium components, molasses (X1), peptone (X3), and MgSO4 (X6) on the yield of viable cells.

The RSM and contour maps for when the number of colonies was used as the response value, are shown in Figures 2A–F. According to Figures 2A,B, when the molasses concentration was constant, with an increase in the peptone concentration, the number of viable bacteria first increased, and then began to decrease at a certain point. When the peptone concentration was constant, with the increase in the molasses concentration, the number of colonies also showed a trend of first increasing and then decreasing. Figures 2C,D shows that when the molasses concentration was constant, the number of *B. velezensis* BHZ-29 colonies first increased and then decreased as the magnesium sulfate concentration increased, and the vertex of the surface was the point with the maximum number of colonies. Similarly, the surface shown in Figures 2E,F reflects the interaction between peptone and magnesium sulfate.

The results when the antibacterial titer was used as the response value are shown in Supplementary Figures S1A–F. It can be seen from Supplementary Figures S1A,B that when the molasses concentration was constant, the antibacterial titer of *B. velezensis* BHZ-29 first increased with the increase of peptone, and then began to decrease when it reached a certain value. When the peptone concentration was constant, with an increase in the molasses concentration, the antibacterial titer of *B. velezensis* BHZ-29 also displayed a trend of first increasing and then decreasing. Supplementary Figures S1C,D shows that when the molasses concentration was constant, the antibacterial titer of *B. velezensis* BHZ-29 first increased and then decreased as the magnesium sulfate concentration increased. The vertex of the surface was the maximum point of the antibacterial titer of *B. velezensis* BHZ-29. Similarly, the surface shown in Supplementary Figures S1E,F reflects the interaction between peptone and magnesium sulfate.

The results when the optimized medium was adjusted to 20.38 g/L molasses, 19.40 g/L peptone, and 3.56 g/L magnesium sulfate are shown in Supplementary Table S3. The average number of colonies was 2.17 × 10^10^ CFU/mL, which was 1.14 times the predicted value, and showed a good degree of fitting with the predicted value. Under these conditions, the bacteriostatic titer was 149.60 mm/mL. When the optimized medium was adjusted to molasses 19.44 g/L, peptone 20.42 g/L, and magnesium sulfate 3.51 g/L, the measured bacteriostatic titer was 153.13 mm/mL, which was 1.01 times the predicted value, and the predicted value had a good fit. The average number of colonies under these conditions was 1.63 × 10^10^ CFU/mL. In summary, the medium selected as the test medium had a molasses concentration of 20.38 g/L, peptone concentration of 19.40 g/L, and magnesium sulfate concentration of 3.56 g/L.

### 3.4 Optimization of the single factor fermentation conditions for *Bacillus velezensis* BHZ-29

**Figure 3:**
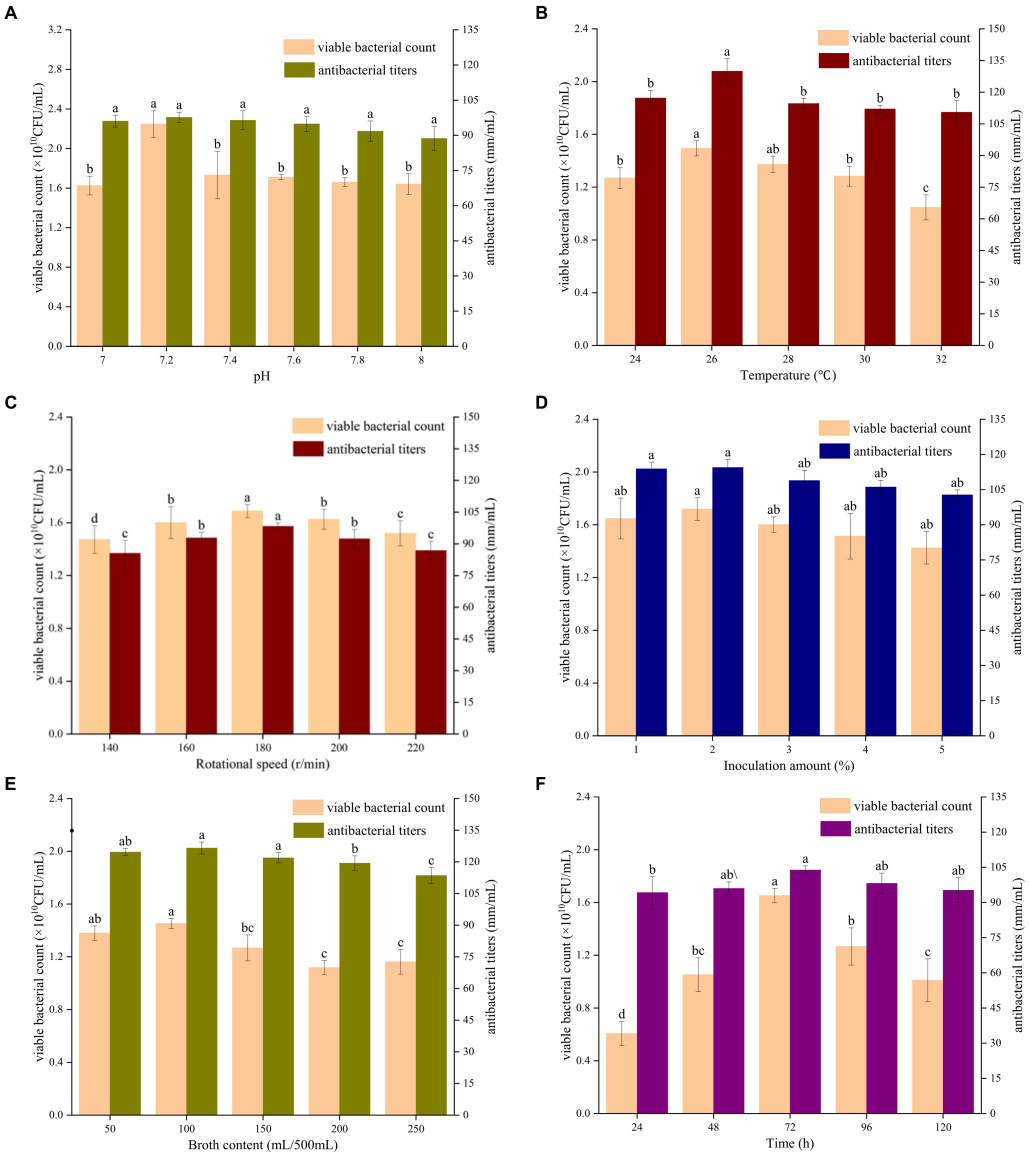
Effects of different initial pH value **(A)**, culture temperature **(B)**, shaking speed **(C)**, amount of inoculant **(D)**, liquid volume **(E)**, and culture time **(F)** on the fermentation of strain BHZ29. Different lowercase letters mean significant difference (*p* < 0.05).

The effects of the initial pH of the medium on the number of viable bacteria and the antibacterial titer of *B. velezensis* BHZ-29 are shown in Supplementary Figure S2. When the initial pH value was in the range of 5–9, *B. velezensis* BHZ-29 was able to grow. The number of viable bacteria and antibacterial titer of *B. velezensis* BHZ-29 were highest when the initial pH value was 7 and 8. Therefore, the initial pH values of 7 and 8 were selected for the next optimization. The results are shown in Figure 3A. With the increase in the pH value, the number of viable bacteria and the bacteriostatic titer displayed a trend of first increasing and then decreasing. When the pH value increased to 7.2, the number of viable bacteria increased to 2.25 × 10^10^ CFU/ml, which was significantly higher than for the other pH values, and the bacteriostatic titer reached 97.70 mm/ml.

As shown in Figure 3B, with an increase in the culture temperature, the number of viable bacteria and bacteriostatic titer displayed a trend of first increasing and then decreasing. When the temperature was 24°C, the viable count of *B. velezensis* BHZ-29 was 1.27 × 10^10^ CFU/ml. When the culture temperature was 26°C, the viable count of *B. velezensis* BHZ-29 was 1.27 × 10^10^ CFU/ml. BHZ-29 had the highest viable count of 1.49 × 10^10^ CFU/mL and highest antibacterial titer of 129.93 mm/ml.

As shown in Figure 3C, with an increase in shaking speed, the number of viable bacteria and antibacterial titer displayed a trend of first increasing and then decreasing. When the shaking speed was 180 rpm, the number of viable bacteria reached a maximum of 1.69 × 10^10^ CFU/ml, and the antibacterial titer reached a maximum of 98.33 mm/ml.

As shown in Figure 3D, with an increase in the amount of inoculant, the number of viable bacteria in the fermentation broth and the antibacterial titer displayed a trend of first increasing and then decreasing, but the trend did not change significantly. When the amount of inoculant was 2%, the number of viable bacteria and the bacteriostatic titer reached the maximum value. At this time, the number of viable bacteria was 1.72 × 10^10^ CFU/ml, and the bacteriostatic titer reached 114.44 mm/ml.

As shown in Figure 3E, with an increase in the liquid volume, the number of viable bacteria displayed a trend of first increasing, then decreasing, and then increasing again, while the antibacterial titer displayed a trend of first increasing and then decreasing. When the liquid volume was 100 mL, the number of viable bacteria and the antibacterial titer reached their maximum values of 1.45 × 10^10^ CFU/ml and 126.56 mm/ml, respectively.

As shown in Figure 3F, with the prolongation of culture time, the number of viable bacteria and the antibacterial titer displayed a trend of first increasing and then decreasing. When the culture time reached 72 h, the number of viable bacteria reached a maximum of 1.65 × 10^10^ CFU/ml, and the bacteriostatic titer was 103.84 mm/ml.

### 3.5 The significant influencing factors of fermentation conditions as determined by an orthogonal test

Based on the results of the single factor test, six factors of temperature (A), rotation speed (B), pH (C), amount of inoculant (D), liquid volume (E), and culture time (F) were selected for an orthogonal test. The test factors and levels are shown in Table 1. The orthogonal test design and results are shown in Table 5. Y1 and Y2 are the number of viable bacteria and bacteriostatic titer, respectively.

**Table 5:** L_18_ (3^6^) Orthogonal experimental design results.

Run numbers	*A*	*B*	*C*	*D*	*E*	*F*	*Y*_1_	*Y*_2_
							(10^10^ CFU/ml)	(mm/ml)
1	1	1	1	1	1	1	0.59	150.10
2	1	2	2	2	2	2	1.96	150.32
3	1	3	3	3	3	3	1.85	161.04
4	2	1	1	2	2	3	3.02	145.70
5	2	2	2	3	3	1	1.95	150.40
6	2	3	3	1	1	2	2.34	152.58
7	3	1	2	1	3	2	1.87	150.51
8	3	2	3	2	1	3	2.49	149.62
9	3	3	1	3	2	1	0.43	149.65
10	1	1	3	3	2	2	1.78	149.73
11	1	2	1	1	3	3	0.84	175.12
12	1	3	2	2	1	1	1.08	150.00
13	2	1	2	3	1	3	3.00	147.87
14	2	2	3	1	2	1	1.76	146.78
15	2	3	1	2	3	2	2.10	151.95
16	3	1	3	2	3	1	0.56	147.70
17	3	2	1	3	1	2	0.87	149.93
18	3	3	2	1	2	3	2.66	146.23
*K*_1_	8.10	10.82	7.85	10.06	10.37	6.37		
*K*_2_	14.17	9.87	12.52	11.21	11.61	10.92		
*K*_3_	8.88	10.46	10.78	9.88	9.17	13.86		
*R*	6.07	0.95	4.67	1.33	2.44	7.49		

The results of the orthogonal test are as shown in Table 5. The changes of F, A, and C (i.e., culture time, temperature, and pH, respectively) had a large influence on the number of viable bacteria, while changes in E, D, and B (i.e., liquid volume, amount of inoculant, and rotation speed, respectively) had little influence. The order of influence on the number of viable bacteria was culture time > temperature > pH > liquid volume > amount of inoculant > rotation speed. The results of the orthogonal test showed that the optimal fermentation conditions were as follows: temperature 26°C, rotation speed 160 rpm, pH 7.2, amount of inoculant 2%, liquid volume 100 ml, and culture time 96 h.

The results of the variance analysis are shown in Supplementary Table S4. Culture time, temperature, and pH had significant effects on the number of viable bacteria. The effects of rotation speed, amount of inoculant, and liquid volume on the number of viable bacteria were not significant. Therefore, the culture time, temperature, and pH, all of which had significant effects on the number of viable bacteria, were selected for the Box–Behnken design experiment.

### 3.6 The Box–Behnken design experiment and analysis of significant influencing factors of fermentation conditions

A regression analysis was performed on the data in Table 6, and the following quadratic polynomial equation was obtained: Y1 = 3.040–0.520A + 0.270C-0.073F + 0.043 AC-0.130AF + 0.200CF-1.170A^2^-0.750C^2^-1.040F^2^ (*R*^2^ = 0.8853, *R*^2^ adjustment = 0.7379).

**Table 6:** The design scheme and test results of the Box–Behnken design.

Run numbers	*A*	*C*	*F*	*Y*_1_	*Y*_2_
				(10^10^ CFU/ml)	(mm/ml)
1	−1	−1	0	1.77	133.63
2	−1	1	0	1.52	132.63
3	1	1	0	0.54	133.60
4	0	0	0	3.23	162.00
5	1	−1	0	0.61	126.73
6	0	−1	1	0.32	160.07
7	0	−1	−1	0.92	124.70
8	1	0	1	0.14	133.60
9	0	0	0	2.22	154.67
10	0	1	1	1.96	166.53
11	−1	0	1	1.40	127.37
12	0	0	0	3.29	143.73
13	−1	0	−1	1.24	132.90
14	1	0	−1	0.49	114.77
15	0	0	0	2.76	153.93
16	0	0	0	3.68	154.53
17	0	1	−1	1.75	132.53

According to the variance analysis results in Supplementary Table S5, the regression model showed that unknown factors resulted in little interference and the model was stable. According to the *F* value, the effect on the number of viable *B. velezensis* BHZ-29 bacteria was: temperature > pH > culture time. According to the *p* value, A2 and F2 were extremely significant (*p* < 0.01), A and C2 were significant (*p* < 0.05), and the other parameters were not significant.

### 3.7 The RSM analysis and determination of the optimal fermentation conditions

**Figure 4:**
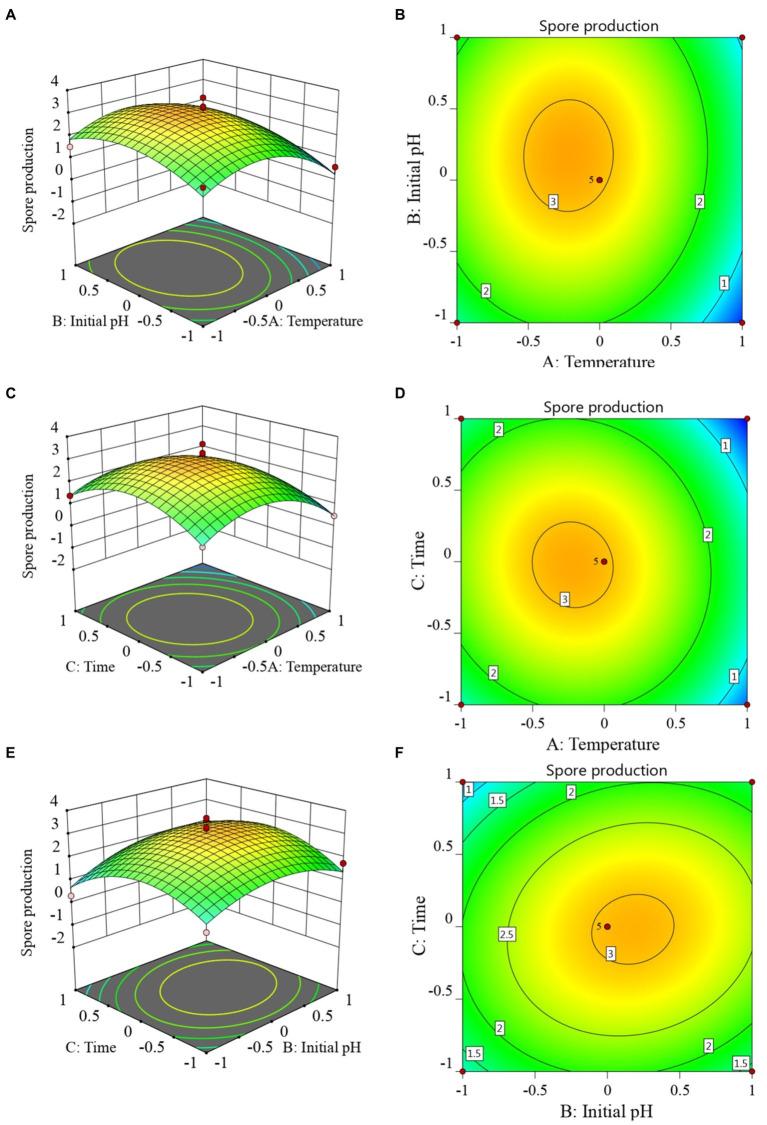
Response surface **(A, C, E)** and contour lines **(B, D, F)** of the interaction among three factors of culture condition, Temperature **(A)**, Time **(C)**, and Initial pH **(E)** on the yield of viable cells.

The RSM diagram and contour diagram between the factors when the number of viable bacteria was taken as the response value are shown in Figures 4A–F. It can be seen from Figures 4A,B that when the temperature was constant, the number of viable bacteria first increased and then decreased with the extension of culture time. When the culture time was constant, with an increase in temperature, the number of viable bacteria also displayed a trend of first increasing and then decreasing. Figures 4C,D shows that when the pH was constant, the number of viable bacteria in *B. velezensis* BHZ-29 first increased and then decreased with the increase of temperature, and the vertex of the surface was the maximum point of the number of viable bacteria. Similarly, the surface of Figures 4E,F reflected the interaction between pH and incubation time. In summary, there was a strong interaction between the factors.

The 3D response surfaces plots were employed to determine the interaction of the fermentation conditions and the optimum levels that have the most significant effect on antibacterial titer. The response surfaces plots based on the model are depicted in Supplementary Figures S3A–F. It is clear from Supplementary Figures S3A that the maximum response of antibacterial titer (156.037 mm) occurred when temperature was at its −0.228075 level. Antibacterial titer increased considerably as temperature increased, indicating that temperature for antibacterial titer has a significant effect on the responses. As the temperature increased, the responses were maximal at 0.725735 level of initial pH and at 0.328792 level of time. The response was also varied at different levels of initial pH and time along the axis, suggesting that there is a considerable interaction between temperature and initial pH, temperature and time, initial pH and initial time (Supplementary Figures S3A–F).

The optimized fermentation conditions were verified, and the results are shown in Supplementary Table S6. The average number of viable *B. velezensis* BHZ-29 bacteria was 3.39 × 10^10^ CFU/mL, which showed a good fit with the predicted value. Under these conditions, the antibacterial titer of *B. velezensis* BHZ-29 was 158.85 mm/ml.

### 3.8 Determination of the inhibitory effect and the control effect of the fermentation broth of *Bacillus velezensis* BHZ-29 before and after optimization on *V. dahlia* in cotton

Based on the above optimization results, an indoor pot experiment was conducted, and the disease index, incidence rate, and agronomic traits of cotton seedlings under each treatment were measured after 30 days. The results are shown in Table 7 and Supplementary Table S7, and revealed that the strain could effectively inhibit the occurrence of cotton *Verticillium* wilt both before and after optimization. The control treatment inoculated only with pathogen (CK1) was most seriously affected. The cotton growth was relatively short, and the leaves were yellow, withered, and the edges were rolled up. These are typical symptoms of *Verticillium* wilt, and the disease index was also high (92.22). *B. velezensis* BHZ-29 had a growth-promoting effect on the cotton seedlings. Compared with the control, agronomic traits (e.g., plant height, stem circumference, root length, root weight, fresh weight and dry weight of aboveground parts of cotton seedlings) were significantly improved after treatment with strain fermentation broth (Supplementary Table S7).

**Table 7:** Biocontrol efficacy of different treatments on cotton *Verticillium* wilt.

Treatment	Inoculation concentration (cfu/ml)	Disease index	Biocontrol efficacy (%)
BHZ-29 + VD	3 × 10^8^ 3 × 10^6^ 3 × 10^5^	14.50 ± 1.80^d^	84.28 ± 1.65^a^
		20.56 ± 4.19^c^	70.71 ± 5.43^ac^
		56.67 ± 7.64^b^	38.55 ± 4.12^b^
CK	–	0.00 ± 0.00^e^	–
VD	–	92.22 ± 2.55^a^	–

Values followed by different letters within a column are significantly different (*P* < 0.05) according to Duncan’s new multiple range test.

**Figure 5:**
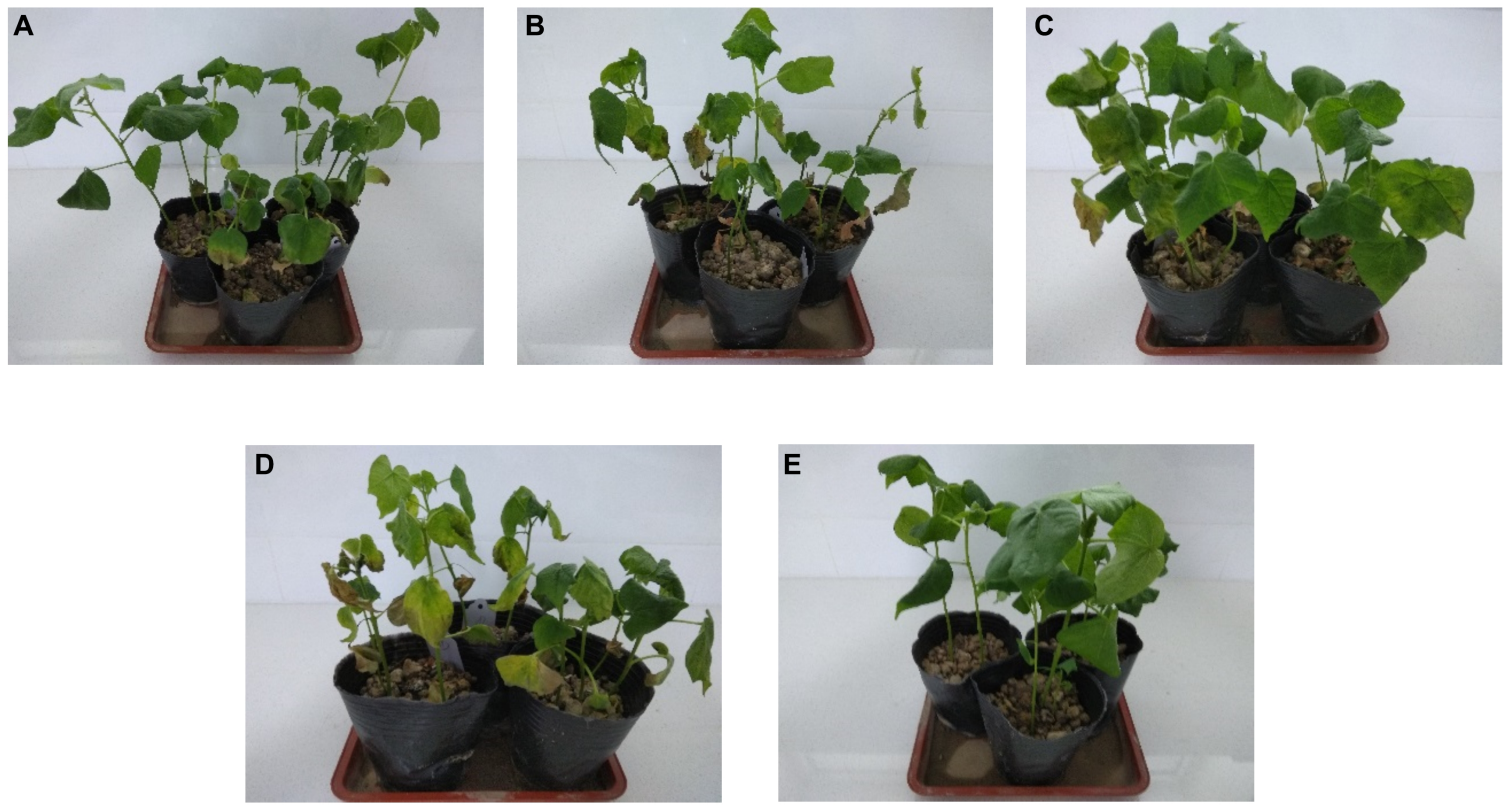
Inhibition effect of four different inoculation concentrations of antagonistic bacteria against *V. dahliae*. **(A)** 2 × 10^8^ cfu/mL; **(B)** 2 × 10^6^ cfu/mL; **(C)** 2 × 10^5^ cfu/mL; **(D)** *V. dahliae*; **(E)** control.

The disease index of the *B. velezensis* BHZ-29 treatment group was significantly different from that of the *V. dahlia* treatment group at the 95% level, indicating that *B. velezensis* BHZ-29 could reduce the disease index of cotton *Verticillium* wilt and had control effect on cotton *Verticillium* wilt (Figure 5). The best effect was obtained at the inoculation concentration of 2 × 10^8^ CFU/mL, with a disease index of 14.50 and control effect of 84.28%. The worst effect was obtained at an inoculation concentration of 2 × 10^5^ CFU/ml, with a disease index of 56.67 and control effect of 38.55%. With the decrease in the inoculation concentration, the disease index of cotton treated with bacteria gradually increased and the control effect gradually decreased, indicating that the higher the inoculation concentration of antagonistic bacteria, the lower the disease index and the better the control effect (Supplementary Table S7).

## 4 Discussion

### 4.1 Harm and control status of cotton *Verticillium* wilt

Xinjiang is the largest cotton production base in China, and cotton *Verticillium* wilt is one of the most important diseases affecting the Xinjiang cotton industry (Xue et al., 2013). Due to perennial continuous cropping, the return of cotton stalks to the field, poor disease resistance, and other unfavorable factors, the occurrence of cotton *Verticillium* wilt in Xinjiang is becoming increasingly serious (Liu et al., 2015a,b), resulting in huge economic losses (Zhu et al., 2023). Biological control has become a hot spot in cotton *Verticillium* wilt research because of its advantages of producing no pollution or residues, not harming humans and livestock, and the lack of resistance to pathogenic bacteria (Liu J. et al., 2023; Liu L. et al., 2023). *Bacillus velezensis* BHZ-29, a highly effective antagonistic bacterium against cotton *Verticillium* wilt, was studied in our laboratory in the early stage of the disease and control effect of 84.18% on cotton *Verticillium* wilt was achieved.

In recent years, the application of *B. velezensis* in agriculture has become increasingly extensive. In previous studies, to take advantage of its antagonistic effects *B. velezensis* has often been isolated from water, soil, air, plant roots, plant surfaces, and animal intestines (Sahal et al., 2023). *Bacillus velezensis* BHZ-29 isolated from healthy cotton plants has a strong adaptability to the natural environment. Previous studies have shown that *B. velezensis* can synthesize lipopeptides, polyketides, bacteriocins, and antibacterial proteins through the polyketide synthase synthesis pathway, ribosome pathway, and non-ribosomal pathway to inhibit the growth of pathogens (Huang et al., 2023). The genome of the *B. velezensis* TSA32 − 1 strain contains genes related to the biosynthesis of lipopeptide antimicrobial substances, such as surfactin, and fengycin family compounds, secondary metabolites known as key factors in biological control (Kim et al., 2022). Similarly, the results of this study showed that *B. velezensis* BHZ-29 bacteria and a sterile fermentation broth had a strong inhibitory effect on *V. dahliae*, and could significantly improve the agronomic traits such as plant height, root length and root weight of cotton seedlings. Similarly to the *B. velezensis* ND (Tang et al., 2023), *B. velezensis* BHZ-29 has a strong biocontrol potential by inhibiting the growth of *V. dahliae*, activating the disease resistance of the cotton system, enhancing the resistance of cotton to *Verticillium* wilt, and increasing cotton yield.

### 4.2 Screening of the fermentation medium and optimization of fermentation conditions for *Bacillus velezensis*

The medium composition has a large influence on the number of viable bacteria and antibacterial products resulting from microbial fermentation, and will determine the ability of microorganisms to inhibit pathogens. After optimizing the culture medium by single factor test and response surface method, the diameter of the inhibition zone of *Bacillus velezensis* P9 fermentation broth was 1.7 times larger than that of the initial inhibition zone (Liu J. et al., 2023; Liu L. et al., 2023). By orthogonal test, the optimal fermentation medium constitutes 40 g/L glucose, 20 g/L corn starch, 25 g/L hot-pressed soybean flour, 3 g/L CaCO_3_. Verification tests suggested the yield of chrysomycin A under optimized conditions reaches up to 3,648 +/− 119 mg/L, which is increased by almost five times (Ni et al., 2021).

In this study, an NB medium was used as the basic medium for the testing of different carbon sources, nitrogen sources, and inorganic salts, and the effects of different medium components on the number of viable bacteria and antibacterial activity of *B. velezensis* BHZ-29 were determined. The optimal medium composition for the colony number fermentation culture *B. velezensis* BHZ-29 was molasses 20.38 g/L, peptone 19.40 g/L, and magnesium sulfate 3.56 g/L, and the populations of the viable organisms were increased to 2.17 × 10^10^ CFU/mL using the optimal fermentation medium. The optimal medium composition for the fermentation culture of *B. velezensis* BHZ-29 was molasses 19.44 g/L, peptone 20.42 g/L, and magnesium sulfate 43.51 g/L, and the bacteriostatic titer in the optimum medium was 131.1 mg/L and the best response was 71.45%, which is more than 28-fold better than the pre-optimized conditions.

### 4.3 Comparison of the effects of fermentation conditions of *Bacillus velezensis* on the number of viable bacteria and antibacterial titer

The optimization of the fermentation conditions of the strain determines the biomass of the strain and the strength of the antibacterial activity (Guo et al., 2014). Optimizing the culture conditions of biocontrol bacteria can increase the amount of bacteria and the yield of antimicrobial substances, thereby improving the biocontrol effect (Naeimi et al., 2020; He et al., 2023). It has previously been reported that the optimal inorganic salt for *B. amyloliquefaciens* HF-01 fermentation is magnesium sulfate, the optimal pH value is slightly acidic to neutral, and the culture temperature is 28°C (Hong et al., 2014), with these values being similar to those reported for *B. velezensis* in this study. This was also similar to the results of this study, but its optimal initial pH was 8.0, while the optimal pH in this study was 7.2, which may be due to the different long-term growth environments of the strains (Hong et al., 2014). The optimal culture conditions of *B. velezensis* BHZ-29 and other biocontrol strains are very different, and the possible mechanisms for this difference need to be further explored.

The culture conditions have a large influence on fermentation, as revealed by their different effects on the number of viable bacteria and antibacterial titer. The fermentation conditions that produced the maximum viable count were likely inconsistent with those that produced the strongest antibacterial potency. When the number of viable bacteria reached 10^9^ CFU/ml or more, antibacterial substances were produced, and therefore the fermentation supernatant had an antibacterial effect (Wu et al., 2020).

### 4.4 Prevention and control of cotton *Verticillium* wilt by *Bacillus velezensis*

*Bacillus velezensis* has long attracted attention as an efficient and safe biocontrol strain that has inhibitory effects on a variety of pathogens (Pandin et al., 2019; Zhang et al., 2022). The biological control of soil-borne cotton *Verticillium* wilt has received increasing attention worldwide (Hasan et al., 2020; Bai et al., 2022; Liu J. et al., 2023; Liu L. et al., 2023; Zhang H. et al., 2023; Zhang L. et al., 2023). The antagonistic bacteria *B. velezensis* BHZ-29 screened in our laboratory can effectively inhibit the growth of *V. dahliae* and increase the peroxidase and superoxide dismutase activities in cotton, indicating that its mechanism of action has antibiosis and induces resistance. Whether there are other mechanisms involved requires further exploration. The highest control effect of cotton treated with *B. velezensis* BHZ-29 on *Verticillium* wilt was 84.18%, and the indoor pot experiment revealed a strong biocontrol potential.

Hasan et al. observed that antagonistic bacteria caused the tip of *V, dahliae* mycelium to expand, hyphae to become abnormal, the number of vacuoles to increase, mitochondria to expand, hyphae to rupture, cytoplasm to overflow, and abnormal microsclerotia (Hasan et al., 2020). Soliman et al. showed that the *Bacillus amyloliquefaciens* could cause the mycelium of the pathogen to break, swell, and deform (Soliman et al., 2022). The results of this study showed that the *B. velezensis* BHZ-29 strain could promote mycelium deformity, overflow, spore perforation, and the depression of *V. dahliae*, resulting in changes to the mycelium and spore morphology.

The induced resistance of biocontrol bacteria could interact with competition, hyperparasitism, antagonism, plant growth promotion, and other mechanisms to exert synergistic effects (Saraf et al., 2014). The reported results of the plate determination of protease, cellulase activity, glucanase activity, and dinitro salicylate method enzyme activity analyses are inconsistent, indicating that the use of the transparent circle diameter as the only indicator of enzyme production capacity is unreliable. Bao et al. (2007) and Jiang and Ding (2015) also reported similar results. With a decrease in the number of bacteria, the enzyme activity also decreased to varying degrees with the prolongation of fermentation time. This may be due to the accumulation of metabolites, apoptosis, and the decreased pH of the fermentation broth with the prolongation of fermentation time, thereby reducing enzyme activity. The *B. velezensis* BHZ-29 strain is rich in cell wall degrading enzymes, especially proteases, which are widely used in feed, leather, and food production (Bao et al., 2012). The antagonistic bacteria *B. velezensis* BHZ-29 can be used as a protease- and other enzyme-producing strain. The *B. velezensis* BHZ-29 strain produces glucanase, protease, and cellulase, with only a low-yield of chitinase. The enzyme activity is greatly affected by the stationary and logarithmic phases of the antagonistic bacteria growth, with a positive correlation between enzyme activity and growth phase (Li R. Q. et al., 2018; Li X. Y. et al., 2018).

## Data availability statement

The original contributions presented in the study are included in the article/Supplementary material, further inquiries can be directed to the corresponding author/s.

## Author contributions

YS: Data curation, Formal analysis, Funding acquisition, Investigation, Visualization, Writing – original draft, Writing – review & editing. XN: Investigation, Resources, Writing – original draft, Writing – review & editing. HY: Investigation, Resources, Writing – original draft. MC: Investigation, Resources, Writing – review & editing. NW: Data curation, Formal analysis, Investigation, Writing – review & editing. HB: Data curation, Resources, Writing – review & editing. FZ: Formal analysis, Investigation, Writing – review & editing. RY: Formal analysis, Investigation, Supervision, Writing – review & editing. KL: Formal analysis, Software, Supervision, Writing – review & editing.
