# Supplementary material for: Optimization of the fermentation media and growth conditions of Bacillus velezensis BHZ-29 using a Plackett–Burman design experiment combined with response surface methodology
Source: Front Microbiol. 2024 Apr 22;15:1355369. doi: 10.3389/fmicb.2024.1355369 (PMC11071168; doi:10.3389/fmicb.2024.1355369)
Supplement: Supplementary file 1 [file Table_1.pdf]

Table S1 | Design and results of the experiments using steepest-ascent method

Run numbers	X_1(g/L)	X_3(g/L)	X_6(g/L)	Y_1 （10^{10} CFU/mL）	Y_2 （mm/mL）
1	10	10	2.5	0.73	149.00
2	15	15	3	1.32	159.60
3	20	20	3.5	1.43	162.45
4	25	25	4	0.81	162.35
5	30	30	4.5	0.62	158.40
6	35	35	5	0.77	157.55
