# Supplementary material for: Optimization of the fermentation media and growth conditions of Bacillus velezensis BHZ-29 using a Plackett–Burman design experiment combined with response surface methodology
Source: Front Microbiol. 2024 Apr 22;15:1355369. doi: 10.3389/fmicb.2024.1355369 (PMC11071168; doi:10.3389/fmicb.2024.1355369)
Supplement: Supplementary file 2 [file Table_2.pdf]

Table S2 | Analysis of variance for response surface quadratic model obtained from experimental results

Source	Sum of squares	*df*	Mean square	*F*-value	*P*(*P*＞*F*)
Model	536.30	9	59.59	12.29	0.0016
X_1	101.75	1	101.75	20.99	0.0025
X_3	46.42	1	46.42	9.58	0.0175
X_6	4.80	1	4.80	0.99	0.3526
X_1X_3	0.04	1	0.04	0.01	0.9302
X_1X_6	9.33	1	9.33	1.93	0.2078
X_3X_6	24.55	1	24.55	5.07	0.0591
X_1^2	118.61	1	118.61	24.47	0.0017
X_3^2	83.47	1	83.47	17.22	0.0043
X_6^2	110.81	1	110.81	22.86	0.0020
Residual	33.93	7	4.85		
Lack of fit	6.28	3	2.09	0.30	0.8229
Pure error	27.64	4	6.91		
Core total	570.23	16			
R^2	0.9405				

coefficient of determination (R^2).
