# Supplementary material for: Optimization of the fermentation media and growth conditions of Bacillus velezensis BHZ-29 using a Plackett–Burman design experiment combined with response surface methodology
Source: Front Microbiol. 2024 Apr 22;15:1355369. doi: 10.3389/fmicb.2024.1355369 (PMC11071168; doi:10.3389/fmicb.2024.1355369)
Supplement: Supplementary file 3 [file Table_3.pdf]

Table S3 | Experimental results of model validation regression

	experimental						predicted
	1	2	3	4	5	mean value	
numbers of viable bacteria ($\times10^{10}$CFU/mL)	2.03	2.30	2.02	2.51	1.99	2.17	1.91
bacteriostatic titers (mm/mL)	156.40	154.73	152.13	153.87	148.53	153.13	151.58
