# Supplementary material for: Optimization of the fermentation media and growth conditions of Bacillus velezensis BHZ-29 using a Plackett–Burman design experiment combined with response surface methodology
Source: Front Microbiol. 2024 Apr 22;15:1355369. doi: 10.3389/fmicb.2024.1355369 (PMC11071168; doi:10.3389/fmicb.2024.1355369)
Supplement: Supplementary file 4 [file Table_4.pdf]

Table S4 | Analysis of variance for orthogonal experimental design

Source of variations	*df*	Sum of squares	Mean square	*F*-value	*P*(*P*＞*F*)	Significant
A	2	3.628	1.814	13.021	0.010	*
B	2	0.076	0.038	0.273	0.772	
C	2	1.852	0.926	6.646	0.039	*
D	2	0.172	0.086	0.618	0.576	
E	2	0.492	0.246	1.767	0.263	
F	2	4.745	2.373	17.032	0.006	*
Pure error	5	0.697	0.139			
Core total	17	65.568				
