# Supplementary material for: Optimization of the fermentation media and growth conditions of Bacillus velezensis BHZ-29 using a Plackett–Burman design experiment combined with response surface methodology
Source: Front Microbiol. 2024 Apr 22;15:1355369. doi: 10.3389/fmicb.2024.1355369 (PMC11071168; doi:10.3389/fmicb.2024.1355369)
Supplement: Supplementary file 5 [file Table_5.pdf]

Table S5 | Analysis of variance for experimental results based on Box-Behnken design

Source	Sum of squares	*df*	Mean square	*F*-value	*P*(*P*＞*F*)	Significan
Model	17.220	9	1.910	6.000	0.0137	*
A	2.150	1	2.150	6.730	0.0357	*
C	0.580	1	0.580	1.820	0.2195	
F	0.043	1	0.043	0.130	0.7256	
AC	0.007	1	0.007	0.024	0.8823	
AF	0.065	1	0.065	0.200	0.6651	
CF	0.170	1	0.170	0.520	0.4946	
A^2	5.800	1	5.800	18.210	0.0037	*
C^2	2.390	1	2.390	7.490	0.0290	*
F^2	4.590	1	4.590	14.400	0.0068	*
Residual	2.230	7	0.320			
Lack of fit	0.970	3	0.320	1.030	0.4676	
Pure error	1.260	4	0.310			
Core total	19.450	16				
R^2	0.8853					

coefficient of determination (R^2).
