# Supplementary material for: Optimization of the fermentation media and growth conditions of Bacillus velezensis BHZ-29 using a Plackett–Burman design experiment combined with response surface methodology
Source: Front Microbiol. 2024 Apr 22;15:1355369. doi: 10.3389/fmicb.2024.1355369 (PMC11071168; doi:10.3389/fmicb.2024.1355369)
Supplement: Supplementary file 6 [file Table_6.pdf]

Table S6 | Experimental results of model validation regression

	experimental						predicted
	1	2	3	4	5	mean value	
numbers of viable bacteria ($\times 10^{10}$CFU/mL)	3.31	3.13	3.42	3.46	3.64	3.39	3.11
bacteriostatic titers (mm/mL)	159.87	157.93	160.60	159.53	156.33	158.85	156.04
