# Supplementary material for: Optimization of the fermentation media and growth conditions of Bacillus velezensis BHZ-29 using a Plackett–Burman design experiment combined with response surface methodology
Source: Front Microbiol. 2024 Apr 22;15:1355369. doi: 10.3389/fmicb.2024.1355369 (PMC11071168; doi:10.3389/fmicb.2024.1355369)
Supplement: Supplementary file 7 [file Table_7.pdf]

Table S7 | Effects of different treatments on biomass of cotton plants after growth for 30 days

Treatment	Inoculation concentration(cfu/mL)	Plant height (cm)	Root length (cm)	The number of root hairs	The number of leaves
BHZ-29	3×10^8	21.24±2.36[a]	7.24±0.73[a]	28.93±1.24[a]	5.97±1.08[a]
	3×10^6	15.74±1.07[b]	5.62±1.65[a]	17.45±1.35[b]	3.89±0.32[c]
	3×10^5	16.32±0.35[abc]	6.22±2.31[a]	18.93±7.61[b]	4.66±0.49[b]
CK	-	13.92±4.15[c]	3.61±0.82b[b]	17.22±2.38[c]	3.42±0.65[c]
VD	-	8.24±1.49[d]	3.43±0.61[b]	16.13±0.75[c]	0.82±0.04[d]

Note: Values followed by different letters within a column are significantly different at $P < 0.05$ level according to Duncan's new multiple range test.
